# Current and Ongoing Developments in Targeting *Clostridioides difficile* Infection and Recurrence

**DOI:** 10.3390/microorganisms12061206

**Published:** 2024-06-15

**Authors:** Wendy Y. Cun, Paul A. Keller, Stephen G. Pyne

**Affiliations:** School of Chemistry and Molecular Science, Molecular Horizons Institute, University of Wollongong, Wollongong, NSW 2522, Australia; keller@uow.edu.au

**Keywords:** *Clostridioides difficile* infection, therapeutics, sporulation, virulence factors, membrane disruption, cationic peptidomimetic, β-lactam, cephamycins

## Abstract

*Clostridioides difficile* is a Gram-positive, spore-forming anaerobic bacterial pathogen that causes severe gastrointestinal infection in humans. This review provides background information on *C. difficile* infection and the pathogenesis and toxigenicity of *C. difficile*. The risk factors, causes, and the problem of recurrence of disease and current therapeutic treatments are also discussed. Recent therapeutic developments are reviewed including small molecules that inhibit toxin formation, disrupt the cell membrane, inhibit the sporulation process, and activate the host immune system in cells. Other treatments discussed include faecal microbiota treatment, antibody-based immunotherapies, probiotics, vaccines, and violet-blue light disinfection.

## 1. Introduction

In 1935, a new Gram-positive, spore-forming anaerobic bacillus species was isolated from the stool of four infants by Hall and O’Toole [1]. It was initially named *Bacillus difficilis* due to the unusual difficulties they encountered during the bacterial isolation and cell culturing process [1,2,3,4]. It was later assigned to the genus *Clostridium* [5] due to its broad phenotypical similarities to the other members of this genus group, although later studies showed it to be distinct enough to be considered as a separate genus [1]. In 2016, a new genus, *Clostridioides*, was created, and *Clostridium difficile* (CD) was reclassified as *Clostridioides difficile*, so it could be differentiated from the previous name but retain sufficient similarities so that the corresponding abbreviated form could remain unchanged [6].

## 2. *Clostridioides difficile* Infection (CDI)

Forty years after the first discovery of *C. difficile*, a team led by Bartlett and co-workers began their investigation of antibiotic-associated diarrhoea. In 1978, they identified toxin-producing *C. difficile* as the major cause of pseudomembranous colitis [7,8,9]. As the bacterium has evolved over time, it has become a threatening pathogen for human society as it is now responsible for a “wide spectrum of diseases”, which are reported as *Clostridioides difficile* infections (CDIs) [10]. This is diagnosed either by clinical evidence of pseudomembranous colitis or by positive laboratory results for toxigenic *C. difficile* in the stool of patients with compatible symptoms. The broad spectrum of CDI symptoms ranges from mild to moderate diarrhoea; severe inflammatory bowel disease; and even fulminant colitis, which could lead to septic shock and possible death [11,12].

As the leading cause of hospital-acquired infections (HAIs), this life-threatening disease with considerably high morbidity and mortality is not only affecting vulnerable and at-risk groups but also the general public [13]. Thus, the impact of CDI has reached all aspects of healthcare delivery. In the United States (US), the estimated annual infections were close to 500,000 cases in 2011, to which 15,000–30,000 deaths were associated [14]. A survey conducted in European acute care hospitals from 2011 to 2012 described *C. difficile* as being the eighth most frequently detected microorganism related to HAIs. In Europe, 123,997 annual CDIs in acute care hospitals were estimated in 2000 with a 3% attributable death rate [15]. Another systematic review on 51 studies of CDI in Asia has revealed a positive rate of 14.8% for tests of CDI among all patients, which is similar to the rate in the US, ranging from 7 to 20%; it has also reported a CDI-related mortality rate of 8.9% in Asia, which is slightly higher than that in the US (6.4%) and Europe (3%) [16]. 

In addition to the significant health impact on patients with CDI, the high cost associated with management of CDI has also placed an economic burden on the healthcare system. An investigation on CDI-associated medical costs in US hospitals from 2005 to 2015 showed that the hospital-onset CDI-attributable cost per case was USD 34,157, which was 1.5 times more than community-onset cost (USD 20,095), and the CDI-attributed cost is an estimated USD 6.3 billion annually [17]. In South Korea, the annual direct and indirect costs for CDI management were estimated as USD 7.6, USD 10.5, and USD 15.8 million in 2009, 2010, and 2011, respectively [18]. A study to estimate the inpatient costs of hospital-acquired conditions in Australian hospitals has found that the annual cost for treatment of enterocolitis due to *C. difficile* was AUD 19,743, which was the third-most costly condition in hospitals, only lower than treatments for post-procedure endocrine/metabolic disorders (AUD 21,827) and MRSA (AUD 19,881) [19].

### 2.1. Pathogenesis of C. difficile

Transmission of CDI can be categorised as endogenous or exogenous. Endogenous CDI originates from *C. difficile* in the carrier state [20], which occurs when the normal gastrointestinal microbiota is disrupted (e.g., when patients receive antibiotic treatment for other bacterial infections) and its efficacy against *C. difficile* is diminished. This creates an intestinal environment favouring growth of *C. difficile*, which eventually leads to CDI [21]. Endogenous *C. difficile* is also an important casual factor in the relapse of CDI after the initial infection and it has been suggested that reinfection is possible when *C. difficile* is not totally eradicated from the bowel after antibiotic treatment [22]. Evidence of *C. difficile* persistence in stool samples was found in patients who had recurrence of CDI after vancomycin treatment. In contrast, patients without a relapse after the treatment tested negative for their stool cultures [22].

Compared to the endogenous infection from the carrier strains, exogenous infection of CDI is more complicated and can be acquired from different sources. With the oral-faecal route as the primary mode of transmission, exogenous CDI refers to the acquisition of *C. difficile* from infected individuals, contaminated healthcare workers, nosocomial sources, or contaminated surroundings [20,23]. It has been proposed that shedding of *C. difficile* spores from patients who were recently treated for CDI is also a possible cause for transmission of *C. difficile*, even after the resolution of diarrhoea [24]. During the treatment of CDI, skin, clothing, and bedding of patients are often contaminated with spores, especially skin scales, which continually renew and shed to provide a source for airborne dispersal of CDI [25]. A study on dissemination of *C. difficile* spores was conducted by Roberts and co-workers in 2006, who successfully isolated and cultured *C. difficile* from the air of a hospital in the UK, providing a plausible explanation for the persistence of CDI in hospitals [26].

In a healthy individual, intestinal microorganisms interact with the host to provide a protective shield against incoming pathogens, including *C. difficile*, keeping the population of harmful bacteria in the gut under control. This *C. difficile*-resistant intestinal environment can be disrupted, commonly through antibiotic therapy during bacterial infections. As the target bacteria are killed by antibiotics, the gut microbiota is also disturbed simultaneously [27,28]. Thus, acquisition of CDI is significantly higher among individuals with diminished intestinal microbiota. Given that *C. difficile* spores can survive in the harsh acidic environment of the stomach, the ingested *C. difficile* spores are able to travel down the small intestine to begin the germination process, which is the transition of inactive spores to metabolically active vegetative cells. Proliferation and colonisation often occur in the descending colon due to its *C. difficile*-favoured anaerobic environment [29]. Infection begins when toxins are secreted from the vegetative cells to induce mucosal inflammation and diarrhoea [23].

### 2.2. Toxigenicity of C. difficile

A diverse range of *C. difficile* types has been disseminated in the literature, with over 800 strains of *C. difficile* reported worldwide to date. However, only the toxin-producing strains, which can cause symptomatic infections, are considered clinically relevant [27]. Clinical and laboratory studies have shown that different toxigenic strains may vary in their pathogenicity and, therefore, differ in their virulence. A study that was conducted in a tertiary care hospital in 1991 discovered that although a *C. difficile*-infected individual typically only carries one type of strain, there were many strains pervasive around the hospital. It was also found that nosocomial diarrhoea was generally associated with multiple-strain infections and that none of the clusters of diarrhoea were caused by only one strain [30]. Thus, the prevalence of this wide diversity of *C. difficile* strains has resulted in a great challenge for controlling transmission and managing outbreaks in hospitals. 

There are two main toxins produced by *C. difficile*—the enterotoxin (toxin A or TcdA) and the cytotoxin (toxin B or TcdB) [31]. TcdA was initially considered as the key virulence factor for *C. difficile* pathogenesis based on the lethal effect of purified TcdA during animal studies, while TcdB showed no disease symptoms [32]. However, like TcdA, TcdB can catalyse glucosylation and deactivation of Rho-GTPases, which affects the actin cytoskeleton and leads to cell death [33]. Thus, TcdB is also responsible for *C. difficile*-induced mucosal inflammation and diarrhoea, especially when the intestinal wall is damaged [9]. 

In recent years, it was found that hypervirulent strains of *C. difficile* produce an additional toxin other than TcdA and TcdB, which is associated with higher mortality rates [34]. This binary toxin, which is referred to as *C. difficile* transferase (CDT), was first identified in a ribotype 027 (RT027) strain of *C. difficile* isolated from a patient with severe pseudomembranous colitis in 1997 and was suggested to be another potential virulence factor [35]. CDT consists of two subunits, the catalytic enzyme subunit CDTa and the binding component subunit CDTb. The C-terminus of CDTb displays a binding domain that interacts with cell surface receptors, which induces accumulation of lipolysis-stimulated lipoprotein receptor (LSR) into lipid rafts, followed by oligomerisation and the binding of the enzyme component CDTa [36]. This allows the translocation of CDTa into the cytosol, which causes ADP-ribosylation of actin and inhibition of actin polymerisation, eventually resulting in the complete destruction of the actin cytoskeleton [37].

### 2.3. Risk Factors of CDI

The majority of the risk factors for developing CDI can be categorised into pharmacological and host-related risk factors [38]. Antibiotic exposure has been considered one of the most important pharmacological risk factors of CDI, especially from broad-spectrum antibiotic agents, which are strongly associated with *C. difficile*-associated diarrhoea (CDAD). When patients receive antibiotic treatments for other bacterial infections, the normal flora of the intestinal microbiota are exposed to these antibiotics, leading to perturbation and disruption of the normal intestinal microflora, allowing *C. difficile* to colonise and cause infections [39]. 

Ampicillin and cephalosporins are the most commonly reported antibiotics that are associated with CDAD [40]. Recent reviews on antibiotic-associated CDAD have been reported [41,42]. A study performed by Denéve and co-workers found that exposure to ampicillin and clindamycin increased the expression of genes encoding for colonisation factors of *C. difficile*, which suggested that these antibiotics can not only disrupt the barrier microbiota in the gut but also facilitate *C. difficile* colonisation [43]. More importantly, it was reported that cephalosporin-implicated CDAD is 40 times more common than that of narrow-spectrum penicillins [40]. However, another study of CDI incidence and cephalosporin usage found that there was no clear association between the incidence and prescription of cephalosporin; as an alternative explanation, it suggests that the key risk factors for CDI are the use of multiple antibiotics and long durations of treatment [44]. This could be related to the inappropriate use of antibiotics in hospitals. A study conducted at two tertiary acute care hospitals in Canada between 2011 and 2012 found that amongst 126 cases of hospital-associated CDI, 45.2% of patients had received inappropriate antibiotic treatments prior to diagnosis [45].

Advanced age is a well-known host-related risk factor for CDI. A study conducted in Finland reported that the incidence of CDI among patients > 64 years old increased sharply from 63/100,000 in the population to 162/100,000 between 1996 and 2004, whereas there was only a slight increase in those who were 45–64 years old, and no increase in patients < 44 years of age [46]. 

Chronic kidney disease (CKD) is another factor that is strongly associated with CDI. It was suggested by Kim and co-workers that CKD is associated with systemic chronic inflammation, which results in immune deficiency and subsequently increases the host’s susceptibility to infection; moreover, gastric acid suppression and microorganism overgrowth are often observed in patients with CKD, which could also cause an increased risk of CDI [31]. Other comorbidities such as inflammatory bowel disease, renal failure, and haematologic cancers are also strongly associated with community-associated CDI [47]. 

### 2.4. Antibiotic Resistance in CDI

The rapid development of antibiotic resistance in *C*. *difficile* has become a major concern for treating CDI. A study of drug resistance in *C. difficile* summarised 15 published reports on antimicrobial-resistant strains of *C. difficile* in Asia, Europe, and the US, from 2001 to 2009, indicating resistance to moxifloxacin increased from 2 to 87%, and resistance to clindamycin increased from 15 to 97% [48]. *C. difficile* is highly capable of adapting to the environment through its metabolic and genomic changes. To date, most strains of *C. difficile* have developed resistance to cephalosporins. The exact mechanism of resistance to cephalosporins is still unknown, but two possible mechanisms have been proposed: (1) via antibiotic-degrading enzymes—β-lactamases—and (2) through modification of target sites—penicillin-binding proteins (PBPs) [49]. Similar to penicillins, cephalosporins inhibit the synthesis of bacterial cell wall peptidoglycans by targeting PBPs. In *C. difficile*, β-lactamases break the β-lactam ring of cephalosporins through hydrolysis to deactivate the drug. Moreover, modification in the active site of PBPs lowers the binding affinity of cephalosporins and consequently mediates drug resistance [50].

Alterations in the antibiotic targets and/or metabolic pathway is a proposed mechanism for *C. difficile* resistance against more commonly used antibiotics other than cephalosporins. Many factors could induce such alterations, and one of the most important factors is the selective pressure from the exposure to antibiotics in vivo [51]. When the concentration of fluoroquinolones (FQs) in vivo is not sufficient to inhibit *C. difficile*, the quinolone-resistance-determining region in two DNA gyrase subunits—GyrA and GyrB—might be triggered and mediate resistance to FQs [51]. Similarly, exposure to rifamycins could lead to mutations in *rpoB* (a gene encoding the β subunit of bacterial RNA polymerase), inducing resistance to rifamycins, especially rifampin and rifaximin. Furthermore, mutations in MurG, a peptidoglycan biosynthesis-associated protein, could also be responsible for the resistance against vancomycin (VAN). The mechanism of metronidazole (MTZ) resistance in *C. difficile* is still not defined, but it was suggested that it might be related to several alterations in metabolic pathways such as iron metabolism and DNA repair [51]. In addition, this selective pressure could also stimulate biofilm formation, which is known to protect bacteria from environmental stresses such as antibiotics. It was also hypothesised that the physiological state of *C. difficile* could be altered into a dormant state within the biofilm matrix, which increases its antibiotic resistance [51].

### 2.5. Management of CDI

Diagnosis of CDI requires both clinical (most commonly diarrhoea in a patient with antibiotic use) and microbiological evidence of the presence of toxin-producing *C. difficile* strains from stool testing [52]. The classification of CDI is based on the severity of the symptoms, which can vary from mild diarrhoea to severe abdominal distension and the occurrence of hypotension. White blood cell count (WBC) and serum creatine level are the two main clinical markers used to classify the severity of the disease. The criteria for each category are outlined in Table 1 [12,53].

As the occurrence of CDI generally follows the disruption of intestinal microbiota—a common side effect after antibiotic treatment—it is important to control the use of antibiotics as a prevention strategy. Antibiotic stewardship programs may be helpful for clinicians with respect to the selection of antibiotics as well as the appropriate dosage and duration of the treatment [54]. Moreover, any antibiotics that predispose the patient to CDI should be discontinued as an ancillary treatment to decrease the risk of recurrence. Guidelines for the treatment of CDI in adults provided by the Infectious Diseases Society of America (IDSA) and the Society for Healthcare Epidemiology of America (SHEA) in 2017 are summarised in Table 2 [24]. Fidaxomicin (FDX) and VAN are strongly recommended for the treatment of the initial episode of CDI. If access to the above agents is limited, MTZ can be used for non-severe CDI only. VAN is also strongly recommended for fulminant CDI. However, although VAN and FDX are recommended for weekly treatment of recurrent CDI, faecal microbiota transplantation is a better option for patients who have had multiple recurrences of CDI and have failed previous antibiotic treatments.

## 3. Treatment of CDI

### 3.1. Treatment of CDI with Antibiotics

#### 3.1.1. Vancomycin (VAN) Treatment

**Figure 1:**
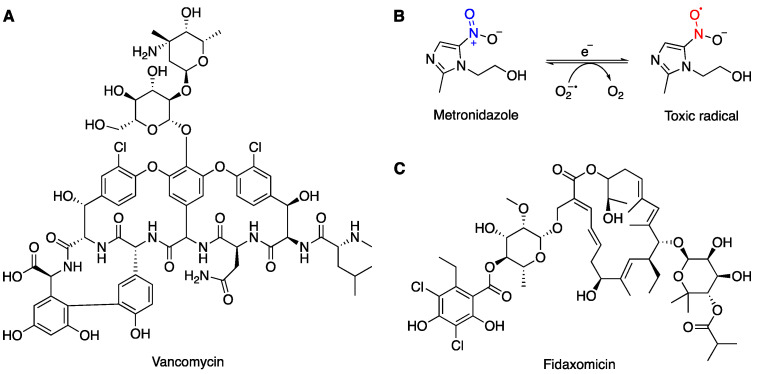
Chemical structures of (**A**) vancomycin (VAN), (**B**) metronidazole (MTZ) and its toxic radical formed in vivo by reduction, and (**C**) fidaxomicin (FDX).

Vancomycin (VAN) (Figure 1A) is a tricyclic glycopeptide antibiotic, which was first isolated in 1957 from the fungus *Streptomyces orientalis* by E. C. Kornfield at Eli Lilly [55]. However, its highly complex structure was not fully resolved until 1981 [56]. This compound was initially labelled as ‘05865’ and was found to be highly effective against Gram-positive bacteria, including penicillin-resistant Staphylococci—an especially concerning clinical pathogen [57]. This compound was then named ‘vancomycin’, derived from the word ‘vanquish’. Shortly after being introduced, VAN was granted fast-track approval by the Food and Drug Administration (FDA) in 1958 [58]. In the early 1980s, its clinical use rose substantially, with a greater than 100-fold increase over the next two decades [57]. VAN is active against most Gram-positive cocci and bacilli, including methicillin-resistant *Staphylococcus aureus* (MRSA)—the most prevalent multidrug-resistant pathogen currently [59,60].

Currently, VAN is still used to treat many bacterial infections, especially CDI. A study conducted in Thailand in 2017 demonstrated that VAN is highly effective against *C. difficile* isolates (n = 105) with minimum inhibitory concentrations (MICs) ranging from 0.06 to 2 μM [61]. Oral administration of VAN is recommended for treating moderate to severe CDI (Table 2). VAN acts by inhibiting the synthesis of bacterial cell walls by binding to the free carboxyl end of peptides containing D-alanyl-D-alanine, which prevents polymerisation of the phosphodisaccharide–pentapeptide lipid complex during Stage II of the cell wall synthesis. Compared to other common antibiotics that are absorbed from the small intestine with low to absent drug concentration in the colon during the therapy, oral administration of VAN showed a high faecal drug concentration and a high rate of recovery. However, the capsule form of VAN is expensive (>USD 1000 for 10 days) and may not be covered by health insurance companies. Moreover, the percentage of VAN-resistant strains of *C. difficile* is gradually increasing. For example, a study conducted in 2015 reported that the RT027 strain with low susceptibility to VAN and MTZ (MIC > 2 mg/L) is the most common strain of *C. difficile* in Israel [62].

#### 3.1.2. Metronidazole (MTZ) Treatment

Metronidazole (MTZ) is a 5-nitroimidazole antibiotic that is derived from azomycin—a natural product that is produced by *Actinobacteria* and *Proteobacteria* spp. [63]. This drug was initially developed in 1959 for the treatment of infection from the parasite *Trichomonas vaginalis*, and its antibacterial activity was accidentally discovered in 1962 after curing a patient with both *Trichomonas vaginalis* infection and ulcerative gingivitis [64,65]. Since the 1970s, the use of MTZ for antibacterial treatment has vastly increased due to its affordable price and broad-spectrum activities against both Gram-negative and Gram-positive anaerobes [66]. As a prodrug, MTZ remains inactive until its nitro group is reduced in vivo after administration (Figure 1B). This reductive activation results in the formation of a toxic radical which, upon regeneration of MTZ, produces oxygen radicals that can induce DNA strand breakage and destabilisation of the DNA helix, which eventually leads to apoptosis [67]. 

MTZ is one of the first-line antibiotic treatments for mild to moderate CDI; it is also used intravenously in combination with VAN for complicated cases, especially in the presence of ileus (Table 1). Although MTZ is less expensive and has a similar efficiency for treating CDI compared to VAN, studies have shown a significantly higher mortality rate in patients who received MTZ [68,69,70]. Furthermore, a study in 2014 found that patients treated with VAN had an approximately 10% higher cure rate than patients who were treated with MTZ for CDI [71]. Therefore, MTZ is more commonly used for patients ≤ 65 years of age with an initial episode of mild to moderate CDI [69]. While MTZ is still effective against most CDI cases, some isolated strains of *C. difficile* showed significantly reduced susceptibility to MTZ [51]. A study reported that 6.3% of MTZ-resistant *C. difficile* strains were isolated from a total of 415 *C. difficile* strains collected from a hospital in Spain from 1993 to 2000 [72]. In recent years, MTZ-resistant strains have been continuously reported from different regions of the world. A surveillance study that was conducted from 2012 to 2014 in Europe showed only 0.11% of investigated strains were resistant to MTZ [73]. In Germany, 2.7% of 1535 *C. difficile* isolates that were obtained between 2014 and 2019 were MTZ-resistant and were almost exclusively RT027 isolates [74]. MTZ-resistant *C. difficile* strains were also reported in Iran, occupying 5.3% of clinical strains from 2010 to 2011 [75]. In China, 15.6% of clinical strains that were collected between 2012 and 2015 were resistant to MTZ; interestingly, this includes one nontoxigenic isolate with MIC > 256 μM [76].

#### 3.1.3. Fidaxomicin (FDX) Treatment

Fidaxomicin (FDX) (Figure 1C) is a narrow-spectrum macrocyclic antibacterial agent that was specially manufactured for the treatment of CDI [77]. Derived from the fermentation of tiacumincin metabolites from actinomycete *Dactylosporangium aurantiacum*, it was approved in 2011 as one of the two front-line treatment drugs for CD [78]. FDX inhibits nucleic acid synthesis by targeting the RNA polymerase of a bacterium at an early step of the transcription initiation pathway [79]. During the binding of holoenzyme to the DNA template to form a complex with RNA polymerase, FDX inhibits the RNA polymerase to prevent the separation of DNA strands, which disrupts messenger RNA synthesis by inhibiting the σ subunit. As the σ subunit differs among bacteria, its narrow-spectral activity avoids cross-resistance with other antibacterial agents during the treatment of CDI. Moreover, oral administration of FDX has shown minimal gut absorption and extremely high faecal concentration levels [78]. Although the resistance of *C. difficile* to FDX has not been reported so far, mutation of *rpoB* and C*D22120* (encoding a homologue of the family of transcriptional regulators MarR) in *C. difficile* clones showed reduced susceptibility to FDX under the selective pressure of FDX use [80,81]. Furthermore, the cost of FDX therapy is > USD 3000 for a 10-day treatment, three times that of VAN therapy [82].

### 3.2. Faecal Microbiota Transplantation (FMT)

Although antibiotic therapies using VAN and FDX are effective against initial episodes of mild to severe CDI, about 20% of patients experience recurrence after the initial episode, and of those, 40–60% experience an additional recurrence [83]. To date, there is still no effective antibiotic therapy available against the recurrence of CDI. Moreover, repeated and extended courses of antibiotics continuously disrupt healthy microbiota in the gut, exposing the vulnerable colon to the spores of *C. difficile* and risking further recurrence. Due to the high costs and high rates of recurrence associated with antibiotic therapies for treating CDI, interest in using faecal microbiota transplantation (FMT) as an alternative therapeutic strategy has been increasing globally [84]. 

The history of FMT dates back to ancient China, where doctors prescribed oral administration of human faecal suspension for patients who suffered from food poisoning or severe diarrhoea in the 4th century. This method was later described in the famous medical book for traditional Chinese medicine—*Ben Cao Gang Mu* (*Compendium of Materia Medica*)—for the treatment of abdominal diseases, referring to this faecal suspension as ‘yellow soup’ [85]. In the early 1940s, German scientists used fresh camel stools to treat soldiers suffering from infectious gastroenteritis in the early 1940s [86]. More recently, the value of ‘healthy faeces’ has been further explored. The first record of employing FMT for the treatment of CDI was a report in 1958. Four patients who were in critical stages of *C. difficile*-induced pseudomembranous colitis had completely recovered after receiving faecal enemas from healthy donors [87].

The general procedure of FMT involves the infusion of faecal matter from a healthy donor into the intestinal tract of the patient, which directly alters the microbial composition of the patient’s gut. The exact mechanisms of the therapeutic effects of FMT have not yet been conclusively identified, but it is believed that the restored gut microbiota outcompetes *C. difficile* for nutrients, which generates an environment resistant to CDI [88,89]. FMT treatments have been found to have high cure rates (87–92%) and are cost-effective and relatively safe treatment options for patients suffering from recurrences of CDI [89]. However, standard procedures reported for FMT are still under development, and none of the treatment protocols have been approved by the FDA [90]. A recent study reported a case of a 3-year-old patient who received FMT treatment after an orthotopic heart transplant, which was correlated with the development of severe mixed rejection with cardiac allograft vasculopathy (CAV) and led to an organ re-transplantation. It was hypothesised that changes in intestinal microflora after FMT treatment could have been related to a possible alteration in the patient’s immune system, leading to the development of cardiac rejection with CAV [91]. In June 2019, the FDA halted several clinical trials of FMT after the death of another patient, which was also linked to FMT [92]. In 2022, Rebyota, a frozen preparation of faecal microbiota, was approved by the FDA for rectal use [93]. 

### 3.3. Alternative Therapeutic Strategies for CDI

While the current antibiotic treatments remain active against most *C. difficile* strains, concerns about the continuously developing antibiotic resistance and high rate of recurrence related to such treatments have encouraged scientists and researchers to develop alternative non-antibiotic or immune-based therapies for treating CDI. Currently, a number of treatments are under development with the potential to replace the more traditional antibiotic treatments.

#### 3.3.1. Antibody (AB)-Based Immunotherapies

As discussed earlier, only toxin-producing strains of *C. difficile* can cause symptomatic infection. Individuals who had previous exposure to nonpathogenic *C. difficile* strains or other related species can possess cross-reacting antigens, inducing the production of antitoxin antibodies [94]. Patients with low antitoxin antibody levels generally experience more severe and prolonged CDI compared to asymptomatic carriers who have higher antitoxin levels [95]. Therefore, antibody-based treatments for CDI have the potential to allow patients to become asymptomatic carriers or experience less severe symptoms with a lower chance of recurrence [96]. A randomised, double-blind, placebo-controlled Phase II study of monoclonal antibodies (mABs) against the two major *C. difficile* toxins (TcdA and TcdB) was performed in 200 patients with symptomatic CDI in 2006 by Medarex. The study found that the recurrence rate among patients who received antibodies in addition to their regular antibiotic treatment was significantly reduced (7%) compared to the placebo group (38%) [97]. Bezlotoxumab—one of the human mABs targeting TcdB of *C. difficile*—has been approved by the FDA for use as a one-time intravenous therapy for CDI to prevent recurrence. A retrospective study in Finland reported that 73% of patients who received bezlotoxumab treatment did not experience a recurrence in the 3 months post-recovery, and furthermore, recurrence was reduced by 63% in patients with severe CDI [98]. However, the potential cost of these antibody immunotherapies has raised concerns for their widespread adoption and, therefore, were suggested to be only used in patients with a high risk of recurring CDI [94].

#### 3.3.2. Probiotics

Probiotics are living organisms that provide health benefits to the host when administered in adequate amounts. As CDI occurs more frequently among individuals with disrupted intestinal microbiota, probiotics with the potential to restore the *C. difficile*-resistant environment in the gut by providing a barrier of low-virulence microorganisms could be an alternative approach for preventing and/or treating CDI [99]. A study in 2017 screened several probiotic bacteria and discovered that a human-derived probiotic, *Lactobacillus reuterin,* was able to inhibit *C. difficile* growth in vitro, with strain 17938 exhibiting inhibition activity comparable to vancomycin; additionally, *L. reueri* showed low susceptibility to the current antibiotics used for treating CDI (VAN, MTZ, and FDX), suggesting its potential use in co-administration with antibiotics in CDI treatment [100]. Other probiotics such as *Saccharomyces boulardii*, *Lactobacillus rhamnosus*, or probiotic mixtures have been reported to possess efficacies against CDI [101]. However, there is insufficient evidence to support the use of probiotic therapy for the treatment of CDI, which requires further investigation and data collection. VE303, a live biotherapeutic product comprising eight commensal Clostridia strains, is currently under development for recurrent CDI. Clinical studies in healthy volunteers indicated that VE303 was safe and well tolerated at the doses tested. VE303 promoted the establishment of a microbiota community known to provide colonisation resistance [102].

#### 3.3.3. Vaccine Development 

Research into immunotherapeutic approaches, such as vaccination, has been undertaken by several research facilities and pharmaceutical companies. One such vaccine candidate was the crude extract of nontoxigenic strains of *C. difficile*. A murine model study that used the membrane fraction of JND13-023 cells (a nontoxigenic strain of *C. difficile*) as a vaccine demonstrated that the vaccinated mice had generated an immune response to produce serum IgG and intestinal fluid IgA, which prevents the adherence of *C. difficile* to Caco-2 intestinal cells in vitro [103]. Vaccine candidates that target the main virulence factors of *C. difficile* (TcdA/TcdB) were reported by Wang et al. in 2018 [104], wherein two constructed chimeric proteins (Tcd169 and Tcd169Fl) demonstrated in vivo protective immunity against *C. difficile*, even with the hypervirulent strain RT027. Recently, Pfizer announced the results from the CLOVER trial—a Phase III randomised, placebo-controlled study—to evaluate the efficacy of the vaccine candidate PF-06425090 in the prevention of CDI among the age group of ≥50 years old. The initial analysis showed 100% vaccine efficacy in preventing medically attended CDI, although the study failed to meet its initial aim of preventing primary CDI [105].

## 4. Sporulation of *C. difficile*

The increase in the incidence and difficulty of treating the recurrence of CDI is fast becoming a crucial clinical issue. Recurrence of CDI can be caused by a relapse from the same strain of *C. difficile* that caused the initial infection or from a reinfection by a new strain [106]. One of the most important reasons for the recurrence is the persistence of *C. difficile* spores, which may persist within the patient after multiple antibiotic therapies. 

### 4.1. Structure of the C. difficile Spore

**Figure 2:**
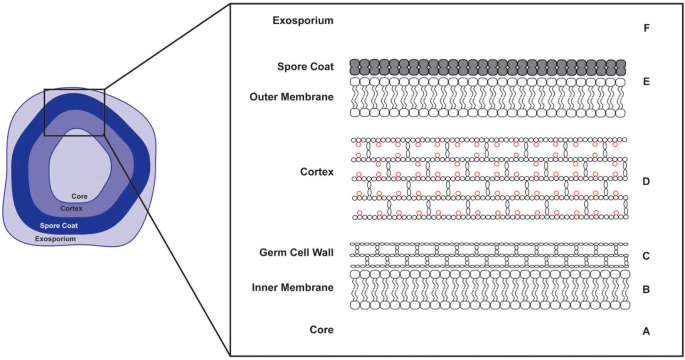
Anatomy of a *C. difficile* spore (reprinted with permission from Ref. [108]. 2018, American Society of Microbiology).

As an anaerobic bacterium, *C. difficile* is highly sensitive to oxygen and is unable to survive outside of the host in its vegetative cell form. Thus, the cells initiate sporulation to produce aerotolerant spores that can persist inside the host and act as a vehicle for transmitting CDI from patient to patient [107]. The structure of a *C. difficile* spore is illustrated in Figure 2 [108].

The unique design of the *C. difficile* spore structure has conferred them with extreme resistance properties, which allow them to survive under variable environmental stresses, including exposure to several common disinfectants. Dormant spores can remain viable for several years and germinate into active vegetative cells when the environment becomes favourable [109]. The centre of the spore consists of a partially dehydrated calcium–dipicolinic acid (Ca-DPA)-filled core, which governs the heat resistance properties of the spore, and the DNA of the pathogen is supercoiled and protected by the bound acid-soluble proteins within the core (Figure 2, A). The core is surrounded by an immobile inner membrane, which has low permeability to many DNA-damaging chemicals such as ethanol (Figure 2, B). The germ cell wall is composed of a peptidoglycan cortex (Figure 2, C), which is surrounded by a thick layer of spore-specific cortex with modified peptidoglycans (Figure 2, D). This modification involves the removal of peptide side chains from 50% of the *N*-acetylmuramic acid (NAM) residues, which allows the conversion of the NAM residues to muramic σ-lactam (MAL). The presence of modified peptidoglycans is crucial for the proliferation of the bacterium, as the abundance of MAL ensures the germ cell wall is rehydrated without being ruptured when the cortex is hydrolysed by cortex lytic enzymes during spore germination. These enzymes are kept in the outer membrane, which is protected by a spore coat (Figure 2, E). The exosporium is the outermost layer of the spore, which contributes to spore dormancy and surface hydrophobicity (Figure 2, F) [108,109,110].

### 4.2. Germination of C. difficile Spores

**Figure 3:**
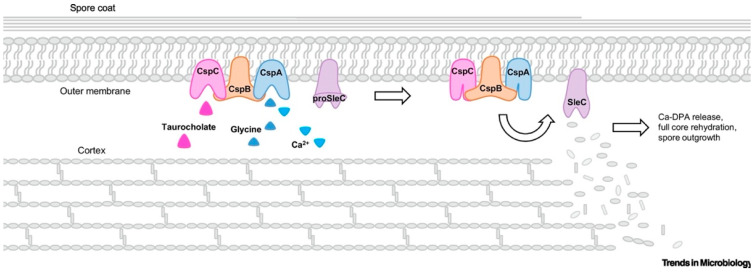
An illustration of the activation of germinant and cogerminant receptors and initiation of germination of *C. difficile* spores (reprinted with permission from Ref. [111]. 2020, Elsevier).

Upon ingestion, *C. difficile* spores can travel through the harsh acidic environment of the stomach and settle in the small intestine, which is the typical site of germination. In spore-forming bacteria, germination is initiated by germinants—nutritious small molecules such as sugar, amino acids, and nucleotides—which interact with specific germination receptors and induce the reactivation of spores into metabolically active vegetative cells [110]. In *C. difficile*, taurocholate—the primary bile salt that is present in the gastrointestinal tract—is a long-recognised germinant, which interacts with a unique receptor—CspC—that is located in the spore coat/outer membrane [111]. A single germinant is not sufficient to induce germination, and therefore a cogerminant is normally required. It has been reported that cholate derivatives and glycine can act as cogerminants to stimulate germination of *C. difficile* [112]. Other substances, such as L-alanine, taurine, and calcium ions, were also found to play a role in cogerminant activity in *C. difficile*. These cogerminants bind to CspA receptors, which helps CspC to transmit germination signals to CspB, which in turn acts as a catalyst to produce active cortex lytic enzyme SleC by cleaving the *N*-terminal region of proSleC—a spore cortex hydrolase. SleC hydrolyses the modified peptidoglycan of the spore cortex, releasing the Ca-DPA and rehydrating the core, which facilitates the outgrowth of *C. difficile* spores into vegetative cells (Figure 3) [111]. CDI is then initiated, and the amount of vegetative cells and the toxin concentration in the colon will typically reach the maximal levels 24 h after the infection [113].

### 4.3. Sporulation Process of C. difficile

During the life cycle of *C. difficile* vegetative cells, sporulation may be initiated in response to environmental stresses and nutrient deprivation and is regulated by the three orphan histidine kinases (CD1579, CD2492, and CD2492) that modulate the master transcriptional regulator Spo0A [113]. Phosphorylation of Spo0A leads to the activation of the gene transcription process, which is regulated by the sporulation-specific sigma factors σF, σE, σG, and σK [114]. Similar to other spore-forming bacteria such as *Bacillus subtilis*, the active site of σF and σG is in the forespore, and σE and σK are active in the mother cell [114]. In addition to Spo0A activation, sporulation of *C. difficile* is also regulated by DNA methylation. A recent study reported that inactivation of the gene that encodes an orphan DNA methyltransferase—a key enzyme that is highly conserved in over 300 global *C. difficile* strains—results in a significant reduction in spore production [115].

**Figure 4:**
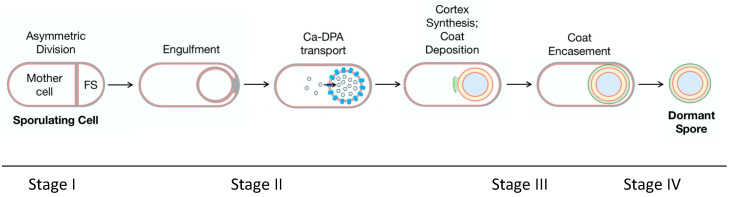
Sporulation process of *C. difficile* (adapted with permission from Ref. [116]. 2019, M. Shen).

The main process of *C. difficile* sporulation is highly conserved in most spore-forming bacteria (e.g., *B. subtilis*), which can be described in four morphogenetic stages (Figure 4) [116]. Following initiation, the first event is the formation of a polar septum, which generates two genetically identical cells—a smaller forespore (FS) and a larger mother cell (Figure 4, Stage I). 

In the next stage, the mother cell engulfs the FS, resulting in the FS being fully contained within the cytoplasm of the mother cell. The coordinated degradation and synthesis of peptidoglycan takes place during the engulfment stage and is followed by the synthesis of Ca-DPA in the mother cell, which is then transported into the core of the FS in exchange for water (Figure 4, Stage II) [116]. As described previously, this partially dehydrated core increases the heat resistance of *C. difficile* spores. 

At Stage III, the spore cortex is synthesised, and the coat and exosporium layers are assembled. Synthesis of the cortex is initiated at the top of the vegetative cell wall, and it is formed between the two membranes that surround the FS. Modification of NAM to MAL (see Section 4.1) during the sporulation is controlled by the activities of two enzymes: CwlD amidase, which removes the peptide side chain from NAM, and PdaA deacetylase, which deacetylates the muramic acid. Coat assembly starts with the polymerisation of coat proteins on the membrane surface that are derived from the mother cell. In *C. difficile*, the inner layer of the coat is formed by two major proteins—SpoIVA and SipL [117,118]. The third morphogenetic protein—CotL—regulates the assembly of the coat, cortex, and exosporium. It has been reported that the loss of CotL in *cotL* mutant spores resulted in a significant reduction in coat proteins and the absence of coat/exosporium layers. These mutants are more sensitive to lysozymes and are defective at the early stage of germination [119]. 

The appearance of the outmost layer (exosporium) varies between *C. difficile*, even within the same strain. Three distinctive morphotypes of exosporium have been identified using scanning electron microscopy (SEM): “smooth”, “bag-like”, and “pineapple-like” [120]. Two cysteine-rich proteins—CdeC and CdeM—are essential for the assembly of *C. difficile* exosporium, although their exact functions in the variability of exosporium remain unknown [121]. 

In the final stage (Figure 4, Stage IV), once the exosporium is assembled and the spore is mature, the mother cell lyses and releases the spore into the surrounding environment [118].

## 5. Current Drug Developments against *C. difficile* Infection

Several potential small-molecule drug targets have been identified against CDI, including those that inhibit toxin formation, disrupt the cell membrane, inhibit the sporulation process, and activate the host immune system. Two review articles on small-molecule inhibitors against CDI were published in 2022 [122,123]. Ideally, a new drug to treat CDI would be selective in inhibiting the growth of *C. difficile* vegetative cells, not disturb the other intestinal microbiota, and prevent reoccurrence of the disease. A recent review article provides a useful summary of new small molecules under patent and in various phases of clinical trials by pharmaceutical companies [124]. 

**Figure 5:**
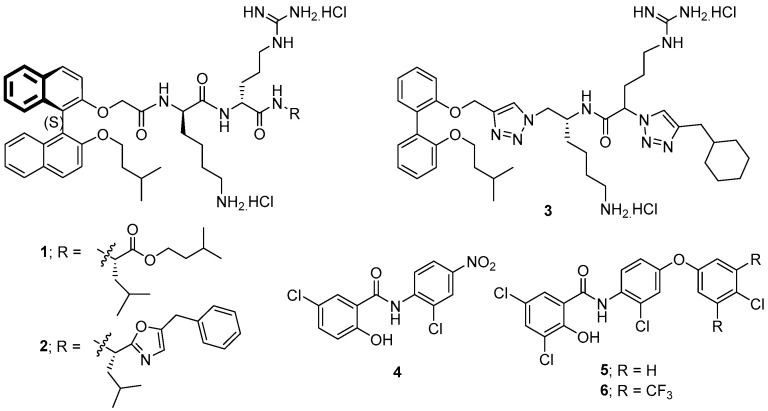
The structures of compounds **1**–**6** that act on the cell membrane of *C. difficile*.

Earlier, our research group developed two novel binaphthyl-based cationic peptidomimetic amphiphiles (**1** and **2**, Figure 5) that showed promising in vitro antibacterial potency against a range of Gram-positive pathogens including strains of *Staphylococcus aureus* resistant to vancomycin, methicillin, and linezolid. Systemic and topical in vivo potency was maintained in mouse models of infection [125]. Further developments around these lead compounds identified other related dicationic peptoids that were active (MIC values of 4–8 μg/mL) against three strains of *C. difficile*, including two problematic ribotype 027 strains, but were also active against other Gram-positive bacteria (*S. aureus* (MIC ≥ 4 μg/mL) and *Enterococcus faecalis* (MIC ≥ 2 μg/mL)) and a Gram-negative bacterium (*Acinetobacter baumannii* (MIC ≥ 4 μg/mL)) [126,127,128,129]. Their broad-spectrum activities would clearly be problematic for their use as therapeutics to treat CDI due to their indiscriminate disruption of intestinal microbiota. Latter studies indicated that the highly hydrophobic binaphthyl moiety of these peptoid derivatives was responsible for their undesirably high haemolytic activities, whereas less hydrophobic biphenyl analogues (e.g., compound **3**) showed better water solubilities, lower haemolytic activities, and enhanced efficacy in an in vivo murine model of CDI [128,129,130]. However, these compounds still displayed broad-spectrum activities. Membrane-disruption assays revealed their likely membrane-active mechanism of action [128,131].

In different studies, the known salicylanilide anthelmintic drugs, closantel, niclosamide (**4**, Figure 5), rafoxanide, and oxyclozanide, were shown to inhibit *C. difficile* growth via a membrane depolarisation mechanism. These known compounds possessed MIC_50_ values against *C. difficile* ranging from 0.06 to 1 μg/mL (vancomycin had an MIC = 1 μg/mL). A library of twenty novel salicylanilide analogues was prepared, with twelve of these compounds having MIC values of <1 μg/mL against two *C. difficile* strains, with compound **5** (Figure 5) being the most potent having an MIC = 0.03 μg/mL. Notably, compound **5** was poorly active against select gut commensals and was nontoxic and nonhaemolytic against mammalian cell lines [132]. 

In a later study, niclosamide **4** was discovered to provide protection to a variety of human cells from both TcdB-induced necrosis and cell-rounding and was equally effective against TcdA and CDT. Niclosamide improved CDI symptoms and, importantly, had no effect on gut microbiota [133]. A latter series of salicylanilides was synthesised, and the most potent analogue **6** was selected through an in vitro inhibitory assay to evaluate its potency in a CDI mouse model [134]. This compound resulted in a reduced recurrence of CDI and reduced disruption of the microbiota in mice when compared to vancomycin. It also reduced bacterial cell and spore counts and thus has demonstrated high potential as a lead for further drug development. 

**Figure 6:**
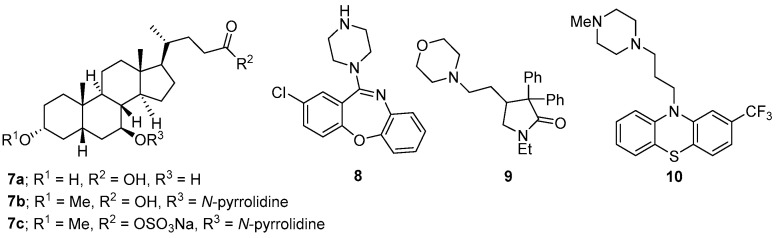
The structures of compounds **7a** and **8**–**10** that regulate the immune system, while **7b**, **7c** inhibit spore germination.

Pretreatment of mice with ursodeoxycholic acid **7a** (ursodiol, Figure 6), a secondary bile acid, significantly altered the bile acid metabolome and host inflammatory transcriptome during CDI. Reduced pathogenesis of *C. difficile* was noted early in the course of the disease. The authors theorised that ursodiol-induced alterations within the intestinal bile acid metabolome resulted in activation of bile acid receptors, which modulated the innate immune response and caused a diminished host inflammatory response that can be detrimental to the host during CDI [135]. Earlier studies revealed that the ursodeoxycholic acid **7a** [136] and its analogues **7b** and **7c** [137] inhibit *C. difficile* spore formation and vegetative growth. The analogues were more potent inhibitors than **7a** and had a more desirable and lower permeability on a Caco-2 model for intestinal epithelial absorption [137].

The known drugs, amoxapine **8** (an antidepresent), doxapram **9** (a respiratory stimulant), and trifluoperazine **10** (an antipsychotic) (Figure 6), were individually demonstrated to alleviate the effects of CDI in mouse models. These drugs were able to reduce *C. difficile* burden and toxin levels through modulation of the host innate immune systems via activation of interleukin 33 [138].

**Figure 7:**
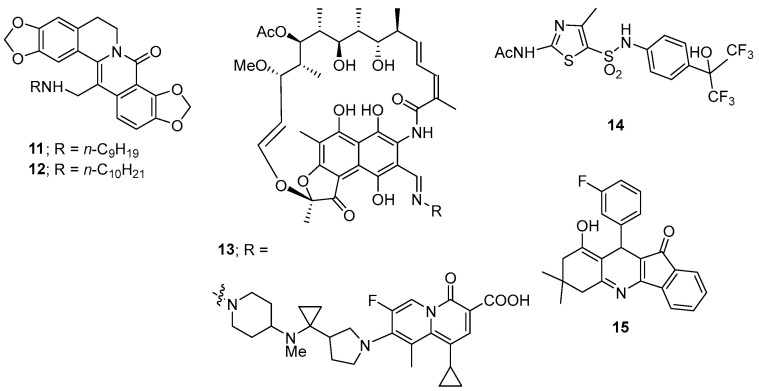
The structures of compounds **11**–**15**.

Twelve 13-[(*N*-alkylamino)methyl]-8-oxodihydrocoptisines were prepared including a series of eight homologues with the *N*-alkyl group ranging from *N*-propyl through to *N*-decyl along with *N*-cyclopropyl, *N*-cyclopentyl, *N*-cyclohexyl, and *N*-homopiperonyl derivatives [139]. Only the more lipophilic *N*-nonyl **11** and *N*-decyl **12** derivatives (Figure 7) were active against *C. difficile* (MIC_50_ values of 7.8 μg/mL) with all other derivatives having MIC_50_ values of 125 μg/mL or greater. These derivatives displayed strong or weak in vivo activating activity towards the transcription factor, x-box binding protein 1 (XBP1, a transcription factor that regulates the expression of genes important to the proper functioning of the immune system and in the cellular stress response), respectively. Significantly, all synthesised derivatives were inactive against *S. aureus* and *E. coli* (MIC > 250 μg/mL).

TNP-2092 (**13**, Figure 7) has antibacterial activity by inhibiting bacterial RNA polymerase, DNA gyrase, and topoisomerase IV. It has poor oral absorption properties, making it a potentially useful agent for treating gastrointestinal infections, with most of the drug recovered in the faeces of treated patients. It has potent antibacterial activities against representative Gram-positive bacteria: *S. aureus*, *Staphylococcus epidermidis, Streptococcus pneumoniase*, *Streptococcus pyogenes* (MIC values as low as 0.015 μg/mL against all bacteria), and *Enterococcus faecalis* (MIC as low as 0.5 μg/mL). It was also highly potent against a *C. difficile* strain (MIC = 0.004 μg/mL). In general, TNP-2092 is far less potent against Gram-negative bacteria. TNP-2092 was administered to rats via single IV bolus (5 mg/kg) or single oral gavage (10 mg/kg). Blood, urine, and faeces samples were analysed after dosage. TNP-2092 showed low systemic exposure after oral administration, and the majority of the drug was recovered in the faeces within 72 h of dosage. TNP-2092 is currently in clinical development for the treatment of symptoms associated with gastrointestinal and liver disorders [140].

It was reported that the thiazole derivative SR1001 (**14**, Figure 7), a known selective RORα (retinoic acid receptor-related orphan receptor alpha) and RORγt inverse agonist (an inverse agonist produces an effect opposite to that of an agonist), blocked T helper 17 cell function and ameliorated recurrent CDI in mice [141].

A small-molecule screen of fifteen compounds identified dihydropyridine **15** (Figure 7) as the most potent compound to confer protection against necrosis in cells caused by the CDI virulence factor TcdB. Compound **15**, and related compounds, disrupt TcdB-induced calcium signalling, resulting in reduced production of reactive oxygen species and subsequent necrosis in cells [142].

**Figure 8:**
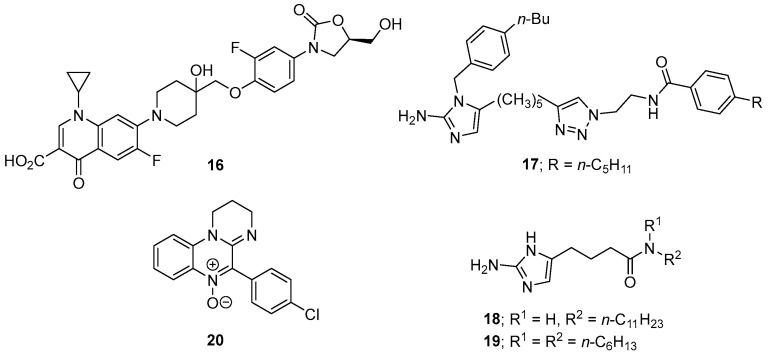
The structures of compounds **16**–**20** that have antibacterial activities against *C. difficile*.

Cadazolid (**16**, Figure 8), having a hybrid structure of a quinolone and an oxazolidinone, is currently in clinical development for the treatment of *C. difficile*-associated diarrhoea (CDAD). Cadazolid has an MIC_50_ of 0.125–0.5 μg/mL against *C. difficile* and showed promising in vivo efficacy in animal (mouse and hamster) models of CDAD. Cadazolid also inhibits formation of spores and the virulence factors TcdA and TcdB [143]. A recent study revealed that cadazolid has potent and selective antibacterial activity against clinically important strains of *C. difficile* and acts by targeting the bacterial protein synthesis machinery. Cadazolid is essentially insoluble in the gastrointestinal tract, a major prerequisite for achieving high drug concentrations at the site of infection as well as minimizing potential side effects due to systemic uptake. Cadazolid has now advanced to Phase III clinical trials [144]. 

A small library of eleven 4-substituted 2-aminoimidazoles was screened for efficacy against *C. difficile*, with compounds **17** (MIC_50_ = 2.5–5 mg/mL), **18** (MIC_50_ = 5 mg/mL), and **19** (MIC_50_ = 5 mg/mL) (Figure 8) having the most potent in vitro activities. Importantly, while compound **19** inhibited *C. difficile* growth, it did not affect the growth of a representative panel of commensal microbes that are associated with a healthy gut microbiota [145].

A series of 4-aryl-1,2-dihydroimindazo [1,2-*a*]quinoxaline 5-oxides was prepared with the 4-chlrophenyl derivative **20** (Figure 8) being the most potent (IC_50_ = 0.25 mg/mL) against two strains of *C. difficile*; this and other analogues were also potent against other anaerobic Gram-positive and Gram-negative bacteria, indicating that **21** may not be suitable to treat CDI. Studies indicated that these compounds may undergo bioreductive activation before becoming antibacterial [146].

Inhibition of sporulation would seem an attractive therapeutic strategy to interrupt the cycle of CDI recurrence and relapse. The sporulation-specific class B penicillin-binding protein (PBP), *Cd*SpoVD, has been identified as a potential anti-sporulation target. *Cd*SpoVD is essential for spore production and is necessary to produce the spore cortex [8], where PBPs, like *Cd*SpoVD, cross-link glycan chains of peptidoglycan [9]. The cephamycin β-lactam antibiotics, cefotetan, cefoxitin, and cefmetazole, inhibit the sporulation process in vitro as well as prevent CDI relapse in in vivo models of disease. These cephamycins bind directly to the active site of recombinant *Cd*SpoVD with high affinity, suggesting that *Cd*SpoVD is the primary molecular target of these drugs [147,148]. These results suggested to us that cephamycin-type analogues could be developed as anti-sporulation agents for the treatment of CDI. However, while cephamycins may reduce spore numbers in the interim, their antibacterial properties may induce gut dysbiosis by disrupting the normal gut microbiota and drive recurrence and relapse of CDI. Furthermore, many circulating *C. difficile* strains currently show resistance to β-lactam antibiotics [10,11]. If cephamycin-type compounds are to be used as anti-sporulation agents then, ideally, they need to be selective for the sporulation process of *C. difficile* and should not cause a disruption to the healthy microbiota of the gastrointestinal tract.

**Figure 9:**
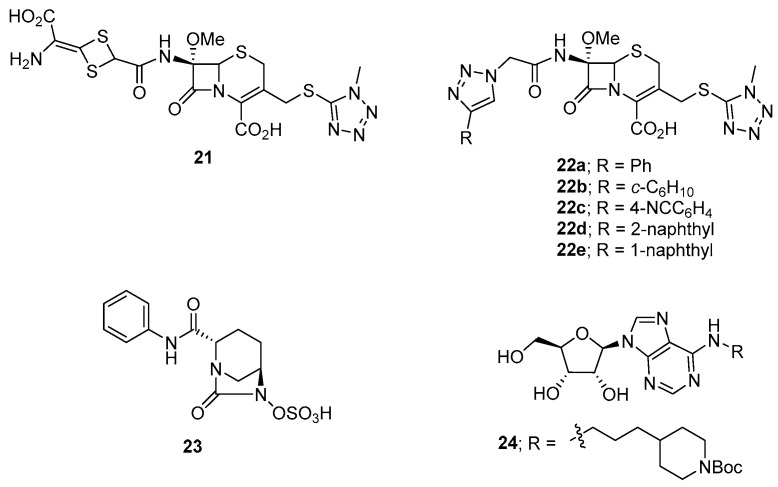
The structures of compounds **21**–**24** that have anti-sporulation activities against *C. difficile*.

Of the aforementioned cephamycin antibiotics, cefotetan **21** (Figure 9) was the most potent and exhibited a 10,000-fold reduction in *C. difficile* sporulation activity at 15 nM [147]. We therefore chose cefotetan **21** as the parent compound for the development of novel anti-sporulation agents targeting *C. difficile* [149]. Based on the versatility of the click reaction to readily prepare 4-substituted 1*H*-1,2,3-triazole derivatives from a common starting azide compound, we decided to prepare a small library of fourteen C-7 α-(4-substituted 1*H*-1,2,3-triazol-1-yl)acetamide cefotetan analogues to test for *C. difficile* sporulation activity and in vitro binding affinity to the target *Cd*SpoVD protein. Of these compounds, **22a**, **22b**, **22c**, **22d**, and **22e** exhibited strong binding affinities (54–90 nM) towards *Cd*SpoVD and potent anti-sporulation activities (>100,000-fold spore reduction) against *C. difficile*, which were 10× more active than the parent compound cefotetan **21**. Furthermore, compound **22a** was tested at 50 µg/mL in a murine model of CDI and exhibited comparable in vivo efficacy to cefotetan (50 µg/mL) after day 4 with the same mice survival rate. Mass spectrometric studies indicated that **21** formed a covalent bond with the target protein with subsequent loss of the thio-*N*-methyltetrazole moiety. Molecular docking studies revealed covalent bonding of **21** to serine-311 of *Cd*SpoVD via nucleophilic ring-opening of the β-lactam ring of **21**. In addition, compounds **22a**, **22c**, and **22d** were selective for *C. difficile* over other common clinical pathogens with no indictive cytotoxicity and haemolytic activity. More importantly, compound **22c** was also selective for the sporulation of *C. difficile* over the vegetative cells of *C. difficile* with an MIC value > 40 μg/mL, suggesting it could be used as an anti-sporulation agent targeting *C. difficile* without the concern for disrupting normal gut flora or inducing antibiotic resistance or relapse of CDI [149].

A recent patent describes several diazabicyclooctane derivatives that inhibit *C. difficile* sporulation in vitro via the inhibition of PBPs essential for spore formation. A representative compound is shown as structure **23**, Figure 9 [150].

*C. difficile* DNA adenine methyltransferase (CamA) mediates *C. difficile* sporulation and colonisation. To discover drug candidates for *C. difficile* that reduce sporulation and minimise intestinal carriage, a series of twenty-three synthetic adenosine analogues were synthesised. Compound **24** (Figure 9) was highlighted for being a potent inhibitor of CamA (IC_50_ = 0.39 μM) and, importantly, was selective for this enzyme over closely related bacterial and mammalian DNA and RNA adenine methyltransferases. Further studies would be required to examine the potential of compound **24** to inhibit sporulation in vivo and its antibacterial activity against *C. difficile* and other bacteria [151].

## 6. Violet-Blue Light Disinfection of Surfaces in Healthcare Facilities

A 2023 study demonstrated that violet-blue light at 405 nm can be used to disinfect surfaces in healthcare facilities and is effective against methicillin-resistant *S. aureus*, vancomycin-resistant Enterococci, and *C. difficile* [152]. However, decontamination of *C. difficile* spores requires a much higher dose of light than permitted in the presence of people, which restricts the use of such methods in combating the recurrence of CDI [152].

## 7. Conclusions

CDI and its recurrence are an ongoing health problem in urgent need of the development of new and inexpensive therapeutic treatments. Current CDI treatments involving antimicrobials often lead to disruption of gut microbiota that leads to recurrence of disease. This is made more challenging to control due to *C. difficile* sporulation. This review article provides background information on CDI and the sporulation process and highlights current research on the discovery of new drugs that have different mechanisms of action to fight against CDI. Of these small-molecule drugs in development, cadazolid (**16**, Figure 8) is well advanced and has now progressed to Phase III clinical trials. TNP-2092 (**13**, Figure 7) also appears to be a promising candidate against CDI. Other drugs highlighted in Figure 5, Figure 6, Figure 7, Figure 8 and Figure 9 often have targets different to the current clinical therapeutics against CDI and may also be potentially useful therapeutics. Inhibition of the sporulation process would seem an attractive therapeutic strategy to interrupt the cycle of CDI recurrence and relapse, with compounds related to, and including, structure 23 (Figure 9) forming part of a recent patent that describes several diazabicyclooctane derivatives that inhibit *C. difficile* sporulation in vitro via the inhibition of PBPs essential for spore formation. However, these compounds and any new small-molecule therapeutics face the challenge of the development of antibacterial resistance. Rebyota, now approved by the FDA for faecal microbiota transplantation (FMT), is an exciting new development that may overcome the initial problems faced by earlier FMT treatments. The probiotic VE303 shows promise in clinical studies in healthy volunteers, while future clinical trials will be required before it can be an approved therapy.

## Figures and Tables

**Table 1:** Guidelines for classification of CDI [12,53].

Severity	Symptoms and Laboratory Evidence
Mild to moderate	Stool frequency of three to five per day WBC ≤ 15,000 cells/mm^3^ Serum creatinine level < 1.5 times of baseline
Severe	Abdominal tenderness Severe colitis (with evidence of clinical examination) WBC ≥ 15,000 cells/mm^3^ Acute rising serum creatinine level > 1.5 times of baseline
Severe and fulminant	Hypotension, ileus, or toxic megacolon Fever (temperature ≥ 38.5 °C) WBC ≥ 35,000 cells/mm^3^ Organ failure
Recurrence	Relapse of CDI within 8 weeks of completion of treatment

**Table 2:** IDSA/SHEA recommendations for the treatment of CDI in adults [24].

Clinical Definition	Recommended Treatment
Initial episode, non-severe	VAN, 125 mg given 4 times daily for 10 days, OR FDX, 200 mg given twice daily for 10 days Alternative treatment option if above agents are unavailable: MTZ, 500 mg, 3 times per day by mouth for 10 days
Initial episode, severe	VAN, 125 mg 4 times per day by mouth for 10 days, OR FDX, 200 mg given twice daily for 10 days
Initial episode, fulminant	VAN, 500 mg 4 times per day by mouth or by nasogastric tube. If ileus, consider adding rectal instillation of VAN Intravenously administered MTZ (500 mg every 8 h) should be administered together with oral or rectal VAN, particularly if ileus is present
First recurrence	VAN 125 mg given 4 times daily for 10 days if MTZ was used for the initial episode, OR Use a prolonged tapered and pulsed VAN regimen if a standard regimen was used for the initial episode (e.g., 125 mg 4 times per day for 10–14 days, 2 times per day for a week, once per day for a week, and then every 2 or 3 days for 2–8 weeks), OR FDX 200 mg given twice daily for 10 days if VAN was used for the initial episode
Second or subsequent recurrence	VAN in a tapered and pulsed regimen, OR VAN, 125 mg 4 times per day by mouth for 10 days followed by rifaximin 400 mg 3 times daily for 20 days, OR FDX 200 mg given twice daily for 10 days, OR Faecal microbiota transplantation

## Data Availability

Data supporting the authors publications cited in this review are available free of charge from the journal’s homepage.
